# Magnetization transfer imaging identifies basal ganglia abnormalities in adult ADHD that are invisible to conventional T1 weighted voxel-based morphometry

**DOI:** 10.1016/j.nicl.2017.03.012

**Published:** 2017-03-30

**Authors:** Arjun Sethi, Edwin Evelyn-Rahr, Nicholas Dowell, Sanjay Jain, Valerie Voon, Hugo D. Critchley, Neil A. Harrison, Mara Cercignani

**Affiliations:** a Clinical Imaging Sciences Centre, Brighton and Sussex Medical School, University of Sussex, Brighton, UK; b Psychiatry, Brighton and Sussex Medical School, University of Sussex, Brighton, UK; c Psychiatry, Addenbrooke's Hospital, , University of Cambridge, UK; d Sackler Centre for Consciousness Science, University of Sussex, Brighton, UK; e Sussex Partnership NHS Foundation Trust, Brighton, UK

**Keywords:** ADHD, Striatum, VBM, MT saturation, T1 weighted MRI, Iron

## Abstract

In childhood, Attention Deficit Hyperactivity Disorder (ADHD) is reliably associated with reduced volume of the striatum. In contrast, striatal abnormalities are infrequently detected in voxel-based morphometry (VBM) neuroimaging studies of adults with ADHD. This discrepancy has been suggested to reflect normalisation of striatal morphology with age and prolonged treatment of symptoms. If so, this would indicate that while striatal abnormalities are linked to symptom expression in childhood, they cannot explain the persistence of these symptoms in adulthood. However, this may not be case. Instead, we hypothesized that the lack of evidence for striatal abnormalities in adult ADHD may reflect poor sensitivity of typical (T1-weighted) neuroimaging to detect subcortical differences. To address this, we acquired both magnetisation transfer (MT) saturation maps optimised for subcortical contrast, and conventional T1-weighted images in 30 adults with ADHD and 30 age, IQ, gender and handedness-matched controls. Using VBM of both datasets, we demonstrate volumetric reductions within the left ventral striatum on MT that are not observed on identically pre-processed T1-weighted images from the same participants. Nevertheless, both techniques reported similar sensitivity to cortical abnormalities in the right inferior parietal lobe. Additionally, we show that differences in striatal iron may potentially explain this reduced sensitivity of T1-weighted images in adults. Together, these findings indicate that prior VBM studies reporting no abnormalities in striatal volume in adult ADHD might have been compromised by the methodological insensitivity of T1-weighted VBM to subcortical differences, and that structural abnormalities of the striatum in ADHD do indeed persist into adulthood.

## Highlights

• Striatal grey matter changes in adult ADHD are robustly observed using VBM of MT maps.
• These changes are not detectable when using typical T1 images in the same subjects.
• Prior negative findings using T1-VBM in adults were thought to reflect therapeutic volumetric normalisation.
• We suggest that such findings reflect the insensitivity of T1-VBM to these changes, not structural normalisation.

## 1 Introduction

Attention Deficit Hyperactivity Disorder (ADHD) is a neurodevelopmental disorder characterised by symptoms of inattention, hyperactivity and impulsivity. Symptoms fully persist into adulthood in around 15% of individuals, with partial persistence observed in around 65% (Faraone et al., 2006). Recently, there has been growing interest in describing the neurobiology of ADHD within this adult population. However, several reports suggest that neurobiological abnormalities characteristic of childhood ADHD are not observed in ADHD persisting into adulthood.

Theoretical accounts of ADHD highlight a central role for deficiencies in dopaminergic signalling within the striatum (Grace, 2001, Sagvolden et al., 2005, Volkow et al., 2005, Castellanos et al., 2006). Aberrant striatal dopamine appears to underpin inattentive (Volkow et al., 2007) and hyperactive/impulsive (Rosa Neto et al., 2002) symptoms, and predicts symptom improvement in response to treatment (Volkow et al., 2012). Moreover, other regional abnormalities detected in ADHD are suggested to be secondary to differences in the striatum (Stanley et al., 2008). Striatal volumetric reductions are also consistently reported in voxel-based morphometry (VBM) studies of childhood ADHD, reinforcing the apparent importance of this structure to the disorder (Nakao et al., 2011, Frodl and Skokauskas, 2012), and offering a non-invasive methodology for investigating striatal abnormalities in ADHD.

While striatal abnormalities are reliably observed in VBM studies of childhood ADHD, similar abnormalities are often not detected in studies of adult patients (Frodl and Skokauskas, 2012). Meta-analyses suggest that this discrepancy may be due to the normalisation of striatal volumes with age and long-term treatment (Nakao et al., 2011). The absence of striatal differences even in symptomatic adult patients also suggests that abnormalities in striatal morphology are not linked to the persistence of ADHD symptoms.

However, methodological explanations could potentially account for the absence of strong empirical support from neuroimaging data linking abnormal structural integrity of the striatum to adult ADHD. For example, less statistically stringent region-of-interest (ROI) based approaches can detect striatal changes in adults with ADHD, even though threshold significance for whole-brain statistical correction is not met (Almeida Montes et al., 2010, Seidman et al., 2011). Importantly, a recent well-powered prospective VBM study also suggests that striatal abnormalities are present in childhood ADHD patients when followed-up as adults, regardless of current diagnostic status (Proal et al., 2011). Interestingly, in this prospective dataset no effects of medication on striatal differences were detected.

Poor methodological sensitivity may represent a previously unexplored explanation for these apparently contradictory findings: meta-analyses typically have fewer and smaller adult studies to draw on, and the T1-weighted neuroimaging sequences used in these VBM studies have poor subcortical contrast. Consequently the automated segmentation of these structures is less accurate. Problematically, inaccuracy of subcortical segmentation using T1-weighted images may also increase with age. Iron accumulates within the striatum over the lifespan (Martin et al., 1998), and has a T1 shortening effect that reduces image contrast (Helms et al., 2009) which may reduce segmentation accuracy in older participants. Moreover, there is recent evidence indicating that ADHD (Cortese et al., 2012, Adisetiyo et al., 2014), and potentially its treatment (Adisetiyo et al., 2014), is also associated with altered brain iron. Previous investigations using T1 weighted images to examine the impact of ADHD status, medication and maturation on striatal volumes may therefore have been systematically biased by these factors. Improving the methodological accuracy and sensitivity of VBM analyses is therefore essential to test whether striatal volumetric differences associated with ADHD do indeed ameliorate with maturation and stimulant treatment, or if the absence of striatal findings in adulthood rather reflects other confounding factors.

Previous data suggest that magnetization Transfer (MT) saturation maps derived from Fast Low Angle Shot (FLASH) 3D multi-parameter provide better subcortical contrast than T1-weighted images traditionally used in VBM (Helms et al., 2009). This is achieved by exploiting the sensitivity of MT to macromolecular protons in myelin, thus yielding an excellent contrast between white and grey matter. Importantly, MT saturation maps are also not affected by the T1 shortening effects of iron that concentrates in the basal ganglia (Helms et al., 2008). MT saturation maps have even been shown to provide contrast between intra-thalamic nuclei (Gringel et al., 2009). Here, we leveraged this improved anatomical accuracy to offer a more definitive examination of the structural changes associated with adult ADHD. We further attempt to reconcile some of the inconsistencies in the ADHD literature, by comparing the sensitivity of MT saturation and conventional T1 maps to detect structural differences in 30 adults with ADHD and 30 age, IQ and gender matched controls. To specifically test for enhanced sensitivity offered by this approach, we compared results to a typical VBM analysis of T1-weighted images obtained in the same participants.

## 2 Materials & methods

### 2.1 Participants

Thirty adult patients with ADHD and 30 healthy control participants were included in the study. Patients with ADHD were recruited from specialist ADHD clinics at Sussex Partnership NHS Foundation Trust (SPFT). All had a DSM-IV confirmed diagnosis of ADHD following comprehensive assessment by a consultant psychiatrist specializing in ADHD management. Assessments included semi-structured clinical interview, self-report CAARS, informant (typically parental) history and completion of Wender-Utah questionnaire, and wherever possible review of school reports. Age, sex and IQ matched controls were recruited though classified advertisements and university mailing lists. All participants were aged 18–65 years. Exclusion criteria included past or current history of any neurological or psychiatric history other than anxiety and/or unipolar depressive disorder currently in remission and past history of significant head injury.

ADHD is associated with increased susceptibility to substance use disorders (Kessler et al., 2006) which have variously been shown to increase (Thompson et al., 2004, Berman et al., 2008, Howell et al., 2013) and decrease striatal volumes (Barrós-Loscertales et al., 2011). To address this potential confound, individuals were screened for current substance abuse, and care was taken by clinicians involved in the study to exclude participants with historical diagnoses of substance use disorders.

Participants gave written, informed consent following full explanation of the experimental procedures. Local and national ethical approvals were obtained from Brighton and Sussex Medical School Research Governance and Ethics Committee (12/131/HAR) and the East of England (Hertfordshire) National Research Ethics Committee (12/EE/0256).

### 2.2 Questionnaires

The Conner's self-report Adult ADHD Rating Scale (CAARS) (Conners et al., 1999) was used to index current ADHD symptom severity. Beck's Depression Inventory (BDI; (Beck et al., 1996)) and the State and Trait Anxiety Inventory (STAI;(Spielberger, 1983)) were used to assess depression and anxiety scores respectively.

### 2.3 Scanning

All neuroimaging was performed on a 1.5T MRI scanner (Siemens Magnetom Avanto, Siemens, Erlangen, Germany) with a body coil used for RF transmission, and a 32-channel head coil for NMR signal reception. MT maps were calculated using a multi-parameter protocol adapted from a 3D multi-echo FLASH sequence (Weiskopf and Helms, 2008). Three co-localised 3D multi-echo flash sequences were acquired in the sagittal plane, each with 1.25 mm^3^ resolution (FOV: 240 × 217.5 mm^2^, 144 partitions). Proton density weighted volumes were acquired with TR = 24 ms, TE from 2.51 to 21.9 ms (eight equidistant bipolar echoes collected), and flip angle (α) of 6°. T1 weighted volumes were acquired with a TR of 19 ms, a TE ranging from 2.51 to 10.82 ms (four equidistant bipolar echoes collected), and a flip angle of 20°. MT weighted volumes were acquired at TR = 30 ms, with the same 4 TEs as the T1-weighted volumes, a flip angle of 12°, and with magnetisation transfer contrast on. These parameters were selected to maximise the contrast between the white matter and the substantia nigra as described elsewhere (Sethi et al., 2013). Standard T1 weighted volumes for VBM were acquired with a magnetization-prepared rapid gradient echo sequence (MPRAGE) (Mugler and Brookeman, 1990) along the axial plane with an isotropic voxel resolution of 1 mm^3^ (FOV: 256 × 240 mm^2^, 192 partitions), with a TE of 3.57 ms, TR of 27.3 ms and a flip angle of 7°. The total acquisition time for the 3 sequences required for MT saturation mapping was approximately 18 min, while the duration of the MPRAGE acquisition was 6 min.

### 2.4 Computation of MT saturation maps

Based on the model of the signal measured in a MT-weighted spoiled gradient echo acquisition (S_MT_) presented in (Helms et al., 2009), the MT saturation, δ, was calculated voxelwise as: δ = (Aα/S_MT_ − 1)R_1_TR − α^2^/2 where: A is the amplitude of the echo at TE, R_1_ is the inverse of T_1_, and α is the imaging flip angle. The resulting maps show a contrast similar to T1-weighted scans and can be entered directly into a VBM pipeline.

### 2.5 Voxel-based morphometry (VBM) pre-processing and analysis

MT saturation and T1 weighted maps were normalized and segmented using the VBM8 toolbox in SPM8 (http://www.fil.ion.ucl.ac.uk/spm/) using a standard pipeline. Images were bias field corrected and segmented into grey matter (GM), white matter (WM) and cerebrospinal fluid (CSF). The segmentation algorithm used adaptive maximum a priori estimations (Rajapakse et al., 1997) and a hidden Markov random field model (Cuadra et al., 2005) to account for partial volume effects (Tohka et al., 2004). Grey and white matter native space segmentations were then normalized to MNI space using a non-linear, high dimensional, diffeomorphic transformation (Ashburner, 2007) to the default DARTEL template supplied with VBM8. Normalized segmentation values were modulated with the nonlinear component of the Jacobian determinants derived from the normalisation. Segmentations were then manually inspected, and smoothed with a 5 mm^3^ full-width half-maximum Gaussian kernel prior to analyses.

Group comparisons were performed according to the General Linear Model (GLM), controlling for age, total intracranial volumes (GM + WM + CSF; derived separately for each modality), and BDI and STAI trait scores. First, we entered both sets of images into a single 2 (imaging sequence) × 2 (diagnosis) second-level mixed measures ANOVA (Flexible Factorial design with subject factor modelled), to test for the presence of a significant interaction, indicating different sensitivity for the 2 modalities ([Control MT > ADHD MT] > [Control T1 < ADHD T1]). In case of significant interaction, we performed 2 separate group comparisons (equivalent to independent sample *t*-tests) for each modality. Treatment effects within the ADHD group were examined in SPM using regression analyses, with length of time on medication (in months) as the variable of interest and age, total intracranial volume, BDI and STAI trait scores as covariates. The results of the whole-brain analyses were accepted as significant at *p* values < 0.05, after false discovery rate (FDR) correction at cluster level (clusters formed with voxel level *p* values < 0.001).

### 2.6 Iron content assessment

Post-hoc assessment of iron content was indexed in the left ventral striatum using R2* maps that have been demonstrated to show good correspondence to brain iron content in post-mortem studies (Langkammer et al., 2010). The left ventral striatum was defined using the mask of Martinez et al. (2003). Maps were calculated from PD maps acquired during the 3D flash sequence by linear fitting of the equation ln(S(t)) = ln(S0) – tR2* to the 8 echo images. In the equation, S is the measured signal, S0 signal at equilibrium, t time at which the signal is sampled (corresponding to the 8 TEs) and R2* the transverse relation rate to be estimated.

## 3 Results

### 3.1 Demographics

Groups were matched for age (mean ± SD: ADHD: 33.7 ± 9.51 years, controls: 32.6 ± 9.54) years, *F*_(1,58)_ = 0.20, *p* = 0.66), IQ (ADHD: 109.0 ± 6.57, controls: 110.1 ± 7.06, *F*_(1,58)_ = 0.40, *p* = 0.53), gender and handedness (Table 1). Mean psycho-stimulant treatment time was 32.7 ± 39.58 months for the ADHD group. As anticipated, the ADHD group scored significantly higher on all CAARS subscales (Table 1). ADHD participants also had significantly higher scores on the BDI (ADHD = 13.7 ± 8.57, controls = 5.6 ± 6.57, *F*_(1,58)_ = 17.01, *p* *<* 0.001) and STAI trait anxiety (ADHD = 53.5 ± 11.04, controls = 36.5 ± 10.71, *F*_(1,58)_ = 36.51, *p* < 0.001).

**Table 1:** Participant demographics and ADHD scores.

Measure	Mean (SD)		*F*	*p*
	ADHD	Controls		
N	30	30	–	–
Male	19	19	–	–
Female	11	11	–	–
Age	33.7 (9.51)	32.6 (9.54)	0.2	0.66
Handedness			–	–
Right-dominant	28	29	–	–
Left-dominant	1	1	–	–
Ambidextrous	1	0	–	–
FSIQ^a^	109.0 (6.57)	110.1 (7.06)	0.4	0.53
CAARS ADHD index	24.0 (5.30)	8.6 (5.01)	133.21	< 0.001
Attention/memory problems	26.7 (5.46)	9.9 (5.67)	123.48	< 0.001
Hyperactivity/motor restlessness	24.4 (6.46)	11.3 (5.68)	68.81	< 0.001
Impulsivity/emotional lability	23.7 (7.36)	7.6 (4.12)	109.13	< 0.001
Problems with self concept	11.2 (4.72)	5.6 (4.45)	22.5	< 0.001
DSM total ADHD score	37.6 (9.03)	12.8 (6.92)	159.66	< 0.001
DSM Inattention	19.3 (4.46)	7.0 (4.55)	125.28	< 0.001
DSM Hyperactivity & impulsivity	18.3 (5.66)	5.7 (3.83)	110.44	< 0.001
BDI	13.7 (8.57)	5.6 (6.57)	17.01	< 0.001
STAI trait	53.5 (11.04)	36.5 (10.71)	36.51	< 0.001

a As estimated by National Adult Reading Test (NART) scores.

### 3.2 Magnetization transfer (MT) saturation maps

**Fig. 1:**
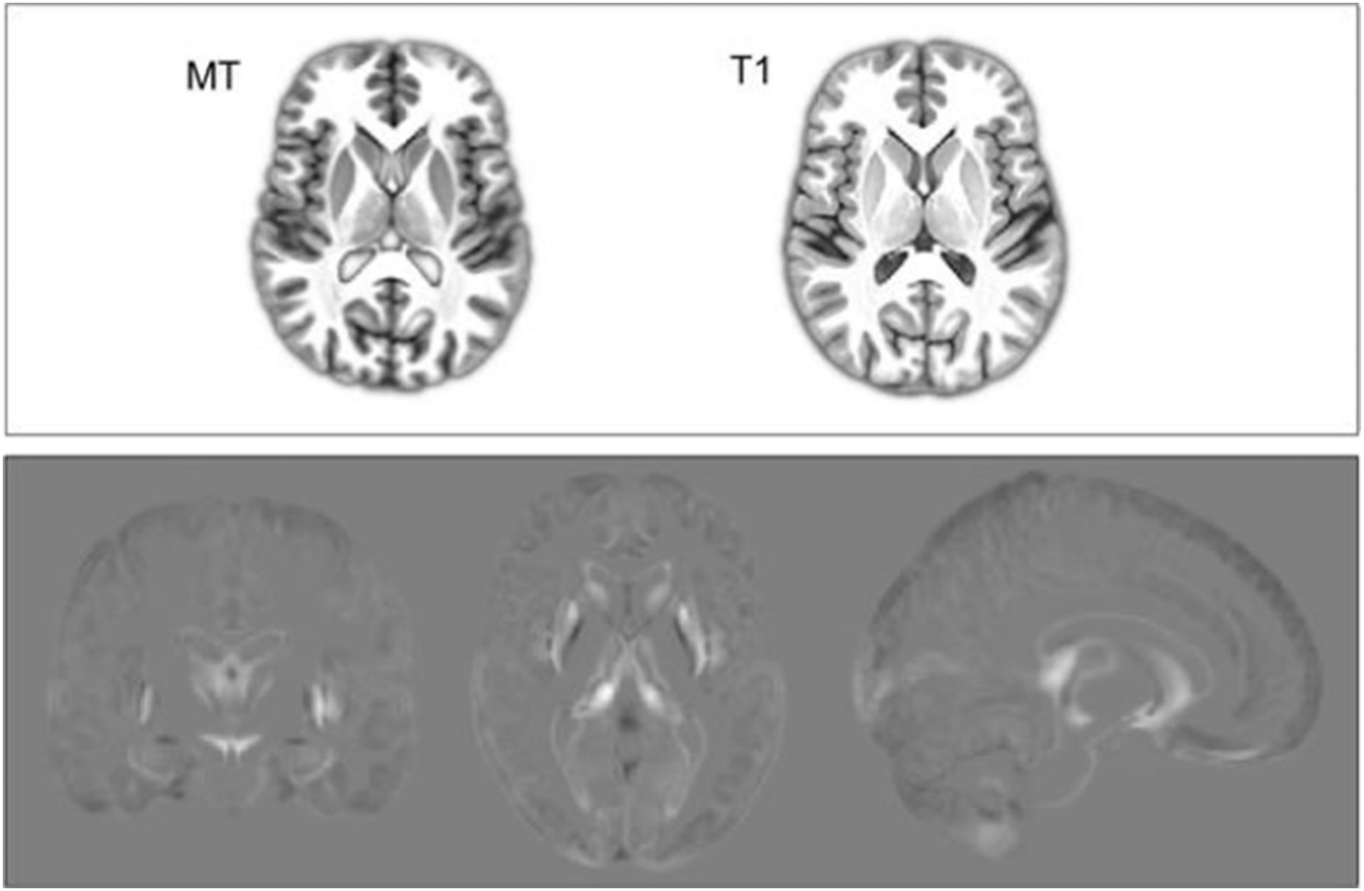
Top: Group averages of MT saturation compared to T1 show enhanced subcortical contrast. Bottom: Difference (MT-T1) in image intensity of segmented averaged maps showing enhanced contrast and segmentation in subcortical regions.

Comparison of MT saturation maps and T1-weighted images highlighted the augmented MT contrast in subcortical regions. The difference of averaged segmentations from each modality also demonstrated enhanced subcortical segmentation accuracy using the MT images (Group average MT saturation map is shown alongside a corresponding T1 group average; Fig. 1).

### 3.3 Voxel-based morphometry (VBM) results

**Fig. 2:**
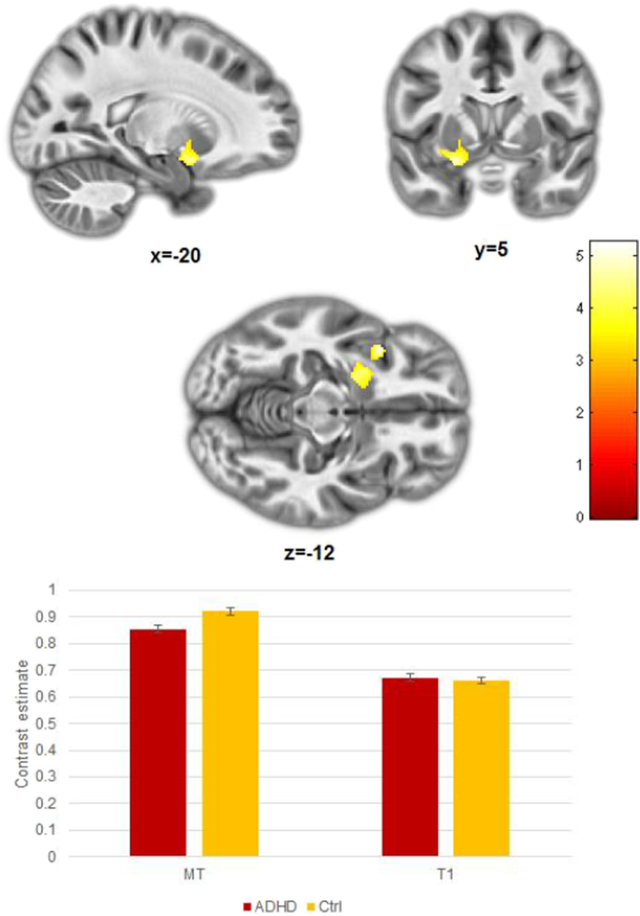
Significant (Control MT > ADHD MD) > (Control T1 < ADHD T1) interaction within the striatum (*p unc.* < 0.001; FDR = 0.001).

The second-level mixed measures ANOVA revealed a significant interaction [(Control MT > ADHD MD) > (Control T1 > ADHD T1)] within a large cluster centred on the striatum and extending into the insula (FDR *p* = 0.001, *k* = 454, Centre: [−27 7–11]; Fig. 2). To investigate this interaction further in a manner consistent with previous studies, we then performed a group comparison on each modality separately.

**Fig. 3:**
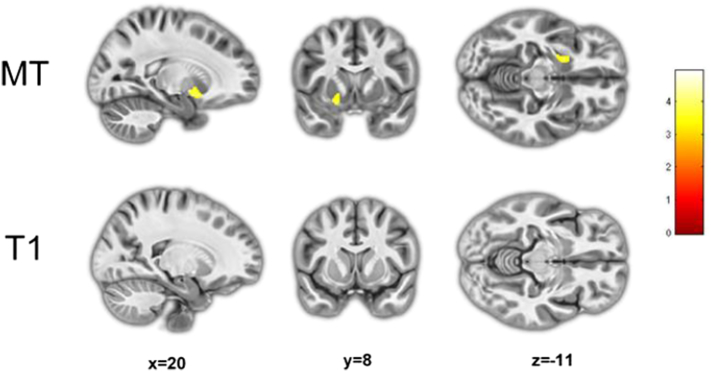
Left ventral striatal volumetric reductions in ADHD detected using MT saturation maps (*p unc*. < 0.001; FDR < 0.05) not detected in T1 weighted volumes.

**Fig. 4:**
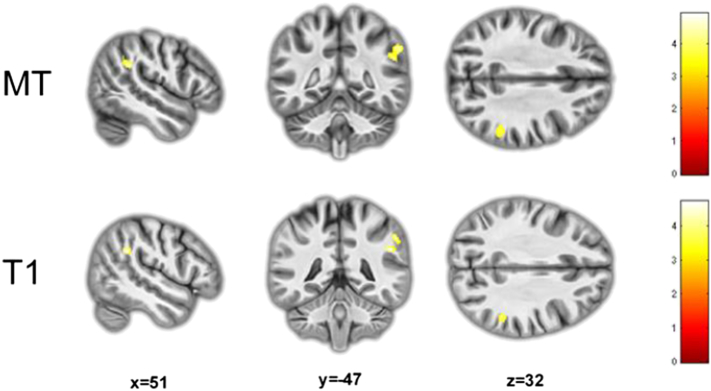
Right inferior parietal volumetric reductions in ADHD detected using MT saturation maps (*p unc.* < 0.001; FDR < 0.05). The same reductions are detected in T1 images (*p unc.* < 0.001) but clusters (*p unc.* = 0.027; *p unc.* = 0.078) did not survive FDR correction.

MT saturation VBM revealed lower grey matter volumes in the left ventral striatum (Fig. 3; Table 2; FDR *p* < 0.05) and right inferior parietal lobe in ADHD compared to controls (FDR *p* < 0.05) (Fig. 4; Table 2), in a region overlapping with the area of significant interaction. By contrast, T1-weighted VBM did not reveal any volumetric alterations within striatal regions (Fig. 3; Table 2) yet did identify the same cortical abnormality within the right inferior parietal lobe (albeit not reaching whole brain FDR cluster level significance; Fig. 4; Table 2). Neither MT nor T1 modality detected any statistically significant increases in regional brain volume in the ADHD group compared to controls. No statistically significant effects of medication were detecting using MT saturation or T1 weighted VBM.

**Table 2:** VBM results: ADHD < Controls.

Region	Peak coordinates	Z	*k* (cluster)	*p unc.* (cluster)	*p* (FDR) (cluster)
MT saturation					
Left ventral striatum	[−20 9 −14]	3.63	189	0.005	0.043⁎
	[−18 8 −8]	3.39			
Right inferior parietal	[59 −45 37]	4.46	294	0.001	0.014⁎
	[51 −42 −28]	4.24			

T1					
Right inferior parietal	[51 −43 28]	4.31	111	0.027	0.377
Right inferior parietal	[56 −43 40]	3.87	67	0.078	0.543

⁎ Significant at whole brain cluster FDR *p* < 0.05.

### 3.4 Iron alterations in the left ventral striatum

To examine whether abnormal iron content may contribute to the methodological insensitivity of T1 to the left hemispheric ventral striatal abnormalities reported, we used a ROI approach to assess R2* as an index for brain iron content. We report increased R2* in ADHD (14.20 ± 2.89) compared to controls (13.26 ± 1.56) in the left ventral striatum (*F*_(1,54)_ = 5.60, *p* = 0.002). Finally, we examined whether these iron differences in ADHD might be influenced by aging or medication use. Left ventral striatal R2* correlated with age (*rho* = 0.37, *p* = 0.045), but not time on medication (*p* > 0.05).

## 4 Discussion

Using MT saturation VBM we observed, contrary to some previous findings, that ventral striatal volumetric reductions persist in adults with ADHD even in patients who have undergone long-term treatment with stimulant medication. Moreover, we show that identically analysed T1 weighted images from the same subjects are insufficiently sensitive to detect these striatal abnormalities. Our data therefore strongly suggest that the absence of striatal volumetric differences observed in previous T1-weighted VBM studies in adults with ADHD likely results from a low methodological sensitivity to subcortical changes, rather than normalisation due to age-related maturation or treatment. This reduced sensitivity of T1-weighted VBM appears to result from poor segmentation accuracy owing to heightened iron content in the striatum. By contrast, both T1-weighted and MT saturation VBM produce similar results in non-subcortical regions such as the inferior parietal cortex.

The observed structural abnormalities in the ventral portion of the striatum reemphasise the importance of the mesolimbic reward system in adult ADHD. Inattentive (Volkow et al., 2007) and hyperactive symptoms (Rosa Neto et al., 2002) are linked to striatal dopaminergic signalling, and striatal reward dysfunction (Scheres et al., 2007) is increasingly seen as a central aetiological component of ADHD (Luman et al., 2010). Our findings suggest that these reward system abnormalities persist well into adulthood. This is in contrast to previous meta-analyses which have shifted focus to cortical regions in adulthood, such as the anterior (Frodl and Skokauskas, 2012) and posterior cingulate (Nakao et al., 2011). The absence of striatal volumetric reductions in adulthood has been suggested to reflect maturational or treatment effects (Nakao et al., 2011). However, our data suggest that this apparent normalisation of striatal volume with age rather reflects difficulties with subcortical segmentation using T1-weighted volumes employed in almost all of these studies. Indeed, we show that MT saturation VBM has significantly improved sensitivity to striatal abnormalities in adult ADHD when compared to T1-weighted images of the same sample.

The present findings do not provide support for maturational or treatment-related normalisation of striatal structure. However, future longitudinal studies will be needed to directly confirm our conclusion. Effects of maturation and treatment on striatal volume are certainly plausible. However, aging and stimulant treatment may induce specific effects on neurobiology that are detectable using MRI aside from grey matter volume. For instance, our data support the association between age (Martin et al., 1998) and ADHD (Cortese et al., 2012, Adisetiyo et al., 2014) (but not medication) and brain iron levels. Future longitudinal work must therefore carefully isolate such factors to accurately study these effects. This is particularly problematic given that one alteration (e.g. brain iron) may interfere with the measurement of another (e.g. volumetric measurement with T1-weighted images) (Helms et al., 2009). Adopting appropriate MR metrics to disentangle these factors and study how they are affected by variables of interest is therefore essential. In particular, this report suggests that T1-weighted imaging of the striatum is inherently biased by other modulators of the MR signal, and is likely to produce confounded results.

MT saturation maps derived from multi-parameter protocols show greatly enhanced subcortical segmentation, owing to their increased sensitivity to myelin and exclusion of T1-shortening effects of iron (Helms et al., 2009). A variety of measures to index proton density, macromolecular/myelin content and iron can also be derived from such protocols. Such approaches therefore not only enhance sensitivity and specificity of subcortical imaging in ADHD, but also offer a richer range of semi-quantitative indices. Given the inherent problems with T1-weighted imaging of subcortical structures, adoption of multi-parameter sequences into large-scale imaging protocols e.g. the ENIGMA consortium may be highly beneficial not just to ADHD research but also studies of Schizophrenia and Bipolar Disorder where subcortical abnormalities are also implicated in the pathophysiology.

Before generalising our results a couple of factors need to be considered. First, this study was conducted at 1.5T and neuroimaging research has increasingly shifted to 3T. T1-weighted image contrast is dependent on field strength. Performance advantages of MT over MPRAGE might therefore differ at 3T. Additionally, MPRAGE is not the only sequence used for high-resolution 3D T1 weighted imaging. Other manufacturers use inversion recovery spoiled GRASS (IR-SPGR). Like MPRAGE, this is based on spoiled fast gradient echoes during inversion recovery. However it differs on acquisition timing and other details, which result in slightly different contrast. Results of automatic segmentation may therefore differ for IR-SPGR. In addition, it is worth noting that scan times for acquisition of full MT saturation mapping (with isotropic resolution of 1.5 mm^3^) are approximately three times longer than for conventional MPRAGE (with isotropic resolution of 1 mm^3^). This must of course be factored in when planning a study.

Future work will also be necessary to determine the extent to which these improvements in subcortical segmentation are also found using other methods (e.g. Freesurfer & FIRST) as the current study and those previous to it (Helms et al., 2008) have exclusively focussed on VBM methods. In particular, whether these improvements are observed in surface-based methods should be tested. However, it should be noted that such benefits would primarily be expected to be observed in subcortical regions striated with white matter and rich in iron.

In addition to ventral striatal abnormalities, we also report volumetric reduction of the right inferior parietal lobe. These findings support the importance of attention networks in ADHD. In particular, these reductions appeared to be localised within the PFm of the supramarginal gyrus (Caspers et al., 2013) which contributes to the ventral attention network (Corbetta and Shulman, 2002). Differences in the ventral attention network in ADHD (McCarthy et al., 2013) may therefore result in abnormal orienting of attention towards salient stimuli (Fox et al., 2006) and subsequent distractibility (Aboitiz et al., 2014). Future multimodal work will be required to assess how these grey matter volumetric abnormalities contribute to dysfunctional ventral attention network connectivity and its neuropsychological consequences.

## 5 Conclusions

The present findings encourage caution in the interpretation of evidence acquired from T1-weighted imaging studies of the basal ganglia. We show that, rather than reflecting age or treatment related normalisation, the failure of previous studies to detect striatal differences is likely to reflect, not their absence but methodological weaknesses of using T1-weighted volumes. We show that these striatal abnormalities in adult ADHD are readily detectable using MT saturation VBM. Adoption of such MT neuroimaging protocols offers the potential for enhanced subcortical segmentation, and increased statistical sensitivity to detect subcortical abnormalities. However, before recommending broader adoption of this approach, our findings would benefit from replication in a larger population. Ultimately, leveraging these benefits may prove essential to characterizing the role of subcortical structures in ADHD, as both age and disorder -related iron content changes can systematically bias analyses using typical T1-weighted images.

## Funding and disclosure

This work was supported by a Wellcome Trust Intermediate Fellowship (WT093881MA) to NAH, Brighton & Sussex Medical School & the Dr. Mortimer and Dame Theresa Sackler Foundation. The authors declare no conflict of interest.
